# Immunohistochemistry-based molecular subtyping of triple-negative breast cancer and its prognostic significance

**DOI:** 10.3389/pore.2023.1611162

**Published:** 2023-05-19

**Authors:** Marisa Leeha, Kanyanatt Kanokwiroon, Suphawat Laohawiriyakamol, Paramee Thongsuksai

**Affiliations:** ^1^ Department of Biomedical Sciences and Biomedical Engineering, Faculty of Medicine, Prince of Songkla University, Hat Yai, Songkhla, Thailand; ^2^ Department of Surgery, Faculty of Medicine, Prince of Songkla University, Hat Yai, Songkhla, Thailand; ^3^ Department of Pathology, Faculty of Medicine, Prince of Songkla University, Hat Yai, Songkhla, Thailand

**Keywords:** immunohistochemistry, breast cancer, prognosis, triple-negative breast cancer, molecular subtype

## Abstract

**Background:** Immunohistochemistry (IHC)-based protein markers representing molecular subtypes are of great value for routine use. This study aimed to evaluate the frequency distributions of the molecular subtypes of triple-negative breast cancer (TNBC) using IHC-based surrogate markers and examined their prognostic value.

**Methods:** Patients with TNBC treated at a university hospital in Southern Thailand were included in this study. Expression levels of androgen receptor, CD8, Forkhead box transcription factor C1, and Doublecortin-like kinase 1 were detected in tumor tissue to classify them into luminal androgen receptor (LAR), immunomodulatory (IM), basal-like immunosuppressed (BLIS), mesenchymal-like (MES), and unclassifiable (UC) subtypes. The association between variables and disease-free survival (DFS) and overall survival (OS) was analyzed using Cox proportional hazards regression.

**Results:** Among the 195 cases of TNBC, the frequency distribution of the IHC-based subtype was as follows: BLIS, 52.8%; LAR, 19.0%; IM, 17.4%; MES, 0.5%; and un-classifiable, 10.3%. BLIS subtype was significantly found in younger ages (mean: 49.6 years) than other subtypes (mean: 51–57.7 years). LAR and BLIS subtypes were significantly associated with poorer OS compared to the IM subtype in univariate analysis, however, only BLIS was significant in multivariate analysis (HR: 3.29, 95% CI: 1.01–10.72). IHC-based subtype was not found to be associated with DFS.

**Conclusion:** This study revealed the differences in the proportion frequency of IHC-based TNBC subtypes in Thai patients compared to other populations. IHC-based molecular subtyping may be beneficial for prognosis. However further refinement of the molecular classification of TNBC is needed for better clinical relevance.

## Introduction

Female breast cancer is the most commonly diagnosed cancer (11.7%) worldwide, with an estimated 2.3 million new cases in 2020. It is also the leading cause of cancer-related death (15.5%) in women [1]. Triple-negative breast cancer (TNBC) is characterized by the lack of estrogen receptor (ER), progesterone receptor (PR), and human epidermal growth factor receptor 2 (HER2), accounting for approximately 15%–20% of all breast cancers [2]. Studies have discovered ethnic variations in TNBC prevalence and clinical behavior, possibly owing to disease heterogeneity and genetic variations among races [3, 4]. Patients with TNBC are younger and have a worse prognosis with a higher relapse rate than non-TNBC [5]. Currently, there is no targeted therapy against TNBC, leaving chemotherapy as the primary treatment option. Hence, there is a need to discover targeted therapies for TNBC to improve the survival rate of patients and enhance their quality of life.

TNBC subtyping based on molecular and clinical features may help to identify potential therapeutic targets and predict prognosis. The most commonly recognized studies on TNBC molecular subtypes include those by Lehmann et al. [6, 7], Burstein et al. [8], and Jiang et al. [9]. Jiang et al. [9] performed multi-omic profiling of 465 Chinese TNBC patients at Fudan University Shanghai Cancer Center (FUSCC) and classified TNBCs into four subtypes: luminal androgen receptor (LAR), mesenchymal-like (MES), basal-like immunosuppressed (BLIS), and immunomodulatory (IM) subtypes. However, using gene expression profiling for molecular subtypes is not practical for routine use because it is time-consuming and expensive and requires fresh frozen tissue. Subsequently, they selected protein markers from each molecular subtype and evaluated their potential use in routine practice by immunohistochemistry (IHC) [10]. They demonstrated that each IHC-based subtype showed substantial differences in clinicopathological features and survival outcomes. However, external validation studies of this IHC-based TNBC subtyping have not yet been reported.

A recent study regarding mutational profiles revealed differences in many frequently mutated TBNC genes in Thai patients compared to that in patients from Western countries [11]. However, there have been no studies on the molecular subtypes of TNBC in Thai patients. In this study, we sought to evaluate the frequency distribution and prognostic significance of the IHC-based molecular subtypes of TNBC in a cohort of Thai patients treated in a university hospital in Southern Thailand. We used IHC-based subtyping proposed by Zhao et al. [10] as it was developed from Chinese patients [9]. This would be the first external validation study of this subtyping algorithm.

## Materials and methods

### Patients and clinical data

This was a retrospective cohort study. The study protocol was approved by the Ethics Committee on Human Research, Faculty of Medicine, Prince of Songkla University (REC. 64-037-4-2). Patients who were diagnosed with TNBC between 2011 and 2019 at Songklanakarind Hospital, a university hospital in Southern Thailand, were included. The inclusion criteria were patients with primary TNBC whose tissue samples before adjuvant chemotherapy and/or radiation therapy were available in our institute. TNBC was identified when ER, PR, and HER2 staining was negative in the tissues using IHC and fluorescence/dual *in situ* hybridization assays. Evaluation of immunoreactivity of ER, PR, and HER2 followed the guidelines of the American Society of Clinical Oncology and College of American Pathologists (ASCO/CAP) [12–14].

Patient data, including age, clinical characteristics, pathological features, treatment, and follow-up information for recurrence, were retrieved from the electronic medical records of the hospital. The first episode of locoregional recurrence or distant metastasis was defined as a recurrence. Locoregional recurrence was defined as tumor recurrence in the chest wall/breast (ipsilateral or contralateral) and four lymphatic drainage regions of the breast (axillary, supraclavicular, subclavian, and internal mammary). Information regarding death was obtained from the Cancer Registry Unit of the faculty, which was updated biannually from the national death registration data.

### Tissue microarray (TMA)

Formalin-fixed, paraffin-embedded (FFPE) tissue samples and hematoxylin and eosin-stained histological slides were retrieved from the archives of the Department of Pathology. A Quick Ray^®^ manual tissue microarray (Unitma, Seoul, Korea) was used for TMA construction. Histological slides were reviewed, and two foci of the representative areas with viable and abundant tumor cells were selected and marked for each case. Then, the corresponding point in the respective FFPE block was marked and cut out using a 2-mm core needle and embedded within the recipient paraffin block. The recipient blocks were then heated at 40°C in a hot-air oven for 15 min. TMA was performed only for excisional biopsied tissue, whereas whole tissue sections were used for core needle biopsy specimens.

### Immunohistochemistry (IHC)

IHC was performed using an automated immunostaining system (BONDMAX; Leica Biosystems, Melbourne, Australia). Briefly, to begin with, the samples were rinsed with xylene for deparaffinization. The slides were rehydrated using increasing concentrations of alcohol and washed with phosphate-buffered saline. Antigens were retrieved and incubated with a bond peroxidase-blocking reagent, followed by primary antibodies against AR (1:200 dilution, Cell Signaling), CD8 (1:500, Cell Signaling), FOXC1 (1:200, Abcam), and DCLK1 (1:100, Cell Signaling Technology). A Bond Polymer Refining Detection Kit (Leica) was used for detection, and 3,3′-diaminobenzidine was used for immunodetection, followed by Mayer’s hematoxylin for counterstaining. We used prostate tissue and tonsil as positive controls for AR and CD8 expression, respectively. TNBC tissues which were tested strongly positive were used as positive control for DCLK1 and FOXC1 expression. The positive control tissues were put simultaneously on all of the examined slides.

### Immunostaining evaluation

Immunostaining was evaluated independently by two authors (M.L and P.T.) who were blinded from clinical data as the percentage of positive cells, irrespective of the signal intensity. For any discordant interpretation, the two authors would study the immunostaining slides together and discuss. Agreement on the positive or negative results of each stain was obtained.

For AR, FOXC1, and DCLK1 expression, the percentage of positive tumor cells over the total number of tumor cells on the slides was estimated. For CD8, stromal CD8^+^ tumor-infiltrating lymphocytes (TILs) were detected in five randomly selected fields (×200 magnification) using digitalized images of full tissue sections. The percentage of stromal CD8^+^ TILs was obtained based on the recommendation of the International TILs Working Group 2014 [15]; that is, the percentage of CD8+TILs was semi-quantitatively estimated over the area of stromal tissue within the borders of the invasive tumor, excluding the TILs outside the tumor boundary, such as tertiary lymphoid aggregation and tumor area with artifacts or necrosis.

For the TMA slides, the average percentage of the two cores was used for further analysis. The cutoff value of positive versus negative protein expression, according to Zhao et al. [10] was used. Tumors positive for AR, DCLK1, and FOXC1 were defined as tumors with ≥ 10% positive cells, whereas CD8-positive tumors were defined as ≥ 20% CD8^+^ TILs.

### Assigning IHC-based molecular subtype

**FIGURE 1:**
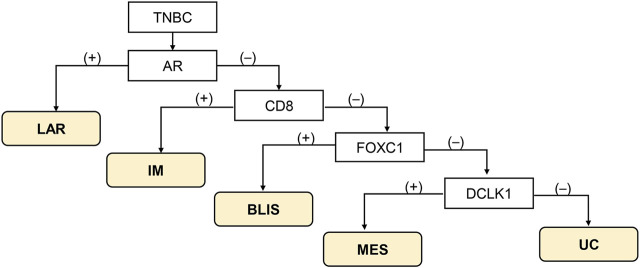
Immunohistochemistry-based classification scheme according to Zhao et al. [10]. Abbreviations: BLIS, basal-like immunosuppressed; IM, immunomodulatory; LAR, luminal androgen receptor; MES, mesenchymal-like; TNBC, triple-negative breast cancer; UC, unclassifiable.

The classification method proposed by Zhao et al. [10] was used (Figure 1). Tumor with AR-positive (+) irrespective of other protein positivity was defined as LAR subtype, AR-negative (−)/CD8+ as IM subtype, AR-/CD8-/FOXC1+ as BLIS subtype, and AR-/CD8-/FOXC-/DCLK1+ as MES subtype. Tumors that could not be classified into any of these subtypes were designated as unclassifiable (UC).

### Statistical analysis

Statistical analysis was performed using R program version 4.0.3 (R Foundation for Statistical Computing, Vienna, Austria). Categorical variables were described by the number of observations and percentages. Continuous variables are represented as mean ± standard deviation (SD). The correlation between clinicopathological characteristics and the TNBC subtypes was calculated using the Fisher exact or Pearson’s chi-square test, as appropriate. Disease-free survival (DFS) was defined as the time interval from the date of diagnosis to the date of first recurrence (local or distant), the date of last known follow-up, or the date of death for those who had no recurrence (censored data). Overall survival (OS) was defined as the time interval from the date of diagnosis to the date of death or the date of the last follow-up if the patients were alive (censored data). OS and DFS were determined using Kaplan-Meier analysis. The Cox proportional hazard regression model was used to determine the prognostic significance of variables. For this analysis, the missing values of clinical stage, chemotherapy and radiotherapy were imputed and replaced by random sampling imputation. IHC-based TNBC subtype and significant variables from univariate analysis were selected for multivariate analysis. Statistical significance was set at a two-sided *p*-value < 0.05.

## Results

### Patient characteristics

During the study period, 198 patients with primary TNBC for whom tissue blocks were available, from the Department of Pathology, Songklanagarind Hospital, between 2011 and 2019 were involved. Three patients had no follow-up information, and a total of 195 TNBC patients were included in the analysis. The mean age of the patients in the cohort was 52.3 years (range: 25–92 years). The patients were grouped into two categories, based on their ages, namely, <50 and ≥50 years, for further analysis. Pathologically, most tumors were invasive ductal carcinomas (invasive breast carcinoma of no special type) (93.3%) and histologic grade 3 (81.5%). At the time of diagnosis, approximately half of the patients were in stage II (47.4%), followed by stage III (27.3%) of the disease. Majority of patients had received mastectomy (61%). Surgery was not performed in 8 patients as they died shortly after biopsy or unfit for surgery. Seventeen patients did not receive chemotherapy due to various reasons including death before treatment (8), denied treatment (3), clinically unfit (3), and non-chemotherapy option for stage I disease (2 patients). Data on treatments were missing in some patients as they transferred to receive treatments at other hospitals.

### IHC-based TNBC subtypes and their clinicopathological characteristics

**FIGURE 2:**
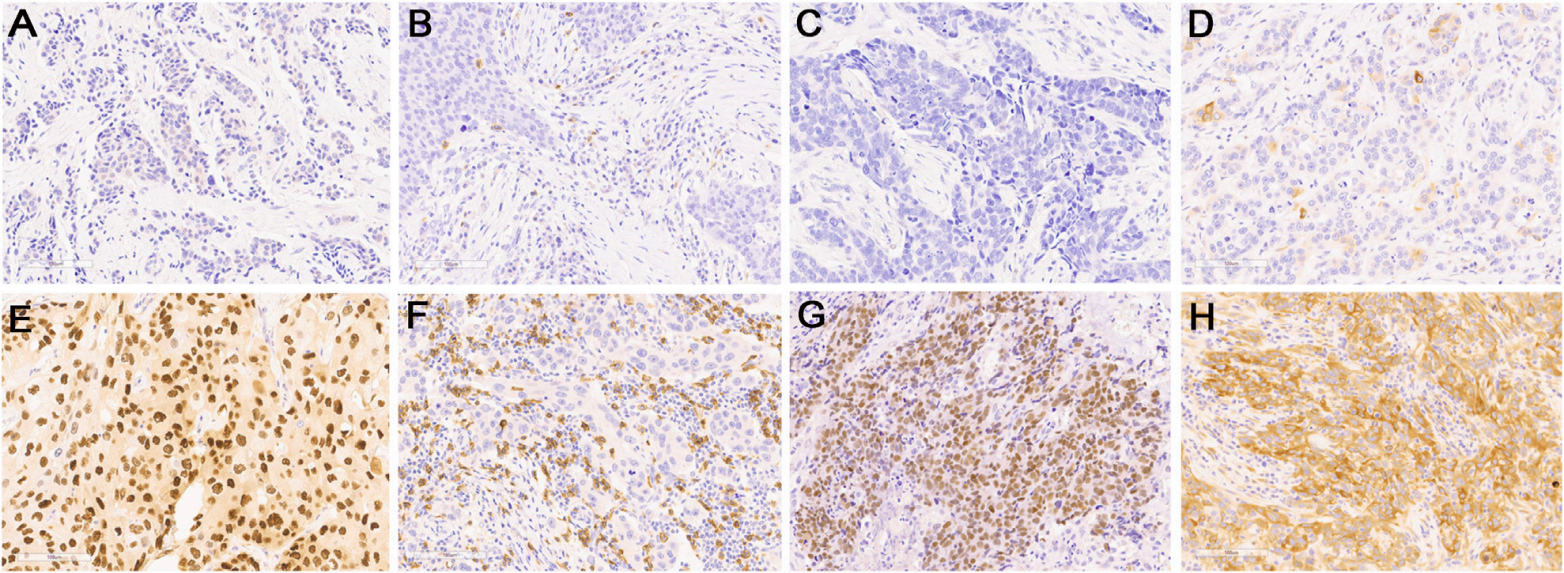
Representative images of immunohistochemical staining for low expression (upper panel) and high expression (lower panel) of protein markers: **(A,E)** AR, **(B,F)** CD8, **(C,G)** FOXC1, and **(D,H)** DCKL1. Original magnification, ×200.

Representative IHC for all proteins is shown in Figure 2. According to IHC-based subtypes, BLIS was the most common subtype (103 cases, 52.82%), followed by LAR (37 cases, 18.97%), IM (34 cases, 17.44%), and MES (1 case, 0.51%). Twenty patients (10.26%) could not be classified and were categorized as UC. The clinicopathological characteristics of the patients according to TNBC subtypes are presented in Table 1. No characteristics, except age, were significantly varied between the subtypes. The BLIS subtype was found majorly in younger patients (mean: 49.6 years) as opposed to the other subtypes (mean: 51–57.7 years). The only one patient with MES subtype was 30 years old with clinical stage III and histologic type of invasive ductal carcinoma, grade 2. The patient received chemo-radiation therapy.

**TABLE 1:** Clinicopathological characteristics of the IHC-based TNBC subtypes.

Variables	LAR	IM	BLIS	MES	UC	*p*-value
	(*n* = 37)	(*n* = 34)	(*n* = 103)	(*n* = 1)	(*n* = 20)	
Age, years						<0.001
Mean (SD)	57.7 (11.7)	55.7 (10.6)	49.6 (12.5)	30	51.0 (10.3)	
<50 years	6 (16.2)	11 (32.4)	61 (59.2)	1 (100)	9 (45)	
≥50 years	31 (83.8)	23 (67.6)	42 (40.8)	0 (0)	11 (55)	
Clinical stage^a^						0.542
I	5 (13.5)	10 (29.4)	22 (21.6)	0 (0)	2 (10)	
II	18 (48.6)	17 (50)	47 (46.1)	0 (0)	10 (50)	
III	11 (29.7)	7 (20.6)	28 (27.5)	1 (100)	6 (30)	
IV	3 (8.1)	0 (0)	5 (4.9)	0 (0)	2 (10)	
Histologic type						
Invasive ductal carcinoma	34 (91.9)	32 (94.1)	97 (94.2)	1 (100)	18 (90)	0.362
Invasive lobular carcinoma	2 (5.4)	0 (0)	1 (1)	0 (0)	2 (10)	
Metaplastic carcinoma	1 (2.7)	1 (2.9)	2 (1.9)	0 (0)	0 (0)	
Other	0 (0)	1 (2.9)	3 (2.9)	0 (0)	0 (0)	
Histologic grade						0.231
1	1 (2.7)	0 (0)	1 (1)	0 (0)	1 (5)	
2	11 (29.7)	5 (14.7)	14 (13.6)	0 (0)	3 (15)	
3	25 (67.6)	29 (85.3)	88 (85.4)	1 (100)	16 (80)	
Lymphovascular invasion						0.419
No	22 (59.5)	27 (79.4)	68 (66)	1 (100)	13 (65)	
Yes	15 (40.5)	7 (20.6)	35 (34)	0 (0)	7 (35)	
Type of surgery^a^						0.245
Conservative surgery	9 (27.3)	13 (40.6)	31 (32)	0 (0)	3 (15)	
Mastectomy	24 (72.7)	19 (59.4)	60 (61.9)	1 (100)	15 (75)	
No surgery	0 (0)	0 (0)	6 (6.2)	0 (0)	2 (10)	
Chemotherapy^a^						0.503
No	4 (11.1)	1 (3.1)	9 (9.1)	0 (0)	3 (15)	
Yes	32 (88.9)	31 (96.9)	90 (90.9)	1 (100)	17 (85)	
Radiotherapy^a^						0.922
No	17 (47.2)	14 (43.8)	44 (44.4)	0 (0)	9 (45)	
Yes	19 (52.8)	18 (56.2)	55 (55.6)	1 (100)	11 (55)	

BLIS, basal-like immunosuppressed; IM, immunomodulatory; LAR, luminal androgen receptor; MES, mesenchymal-like; UC, unclassifiable; SD, standard deviation.

^a^ Number of missing data: clinical stage (1); chemotherapy (7); radiotherapy (7), type of surgery (12).

### Disease-free survival and overall survival of the patients

**FIGURE 3:**
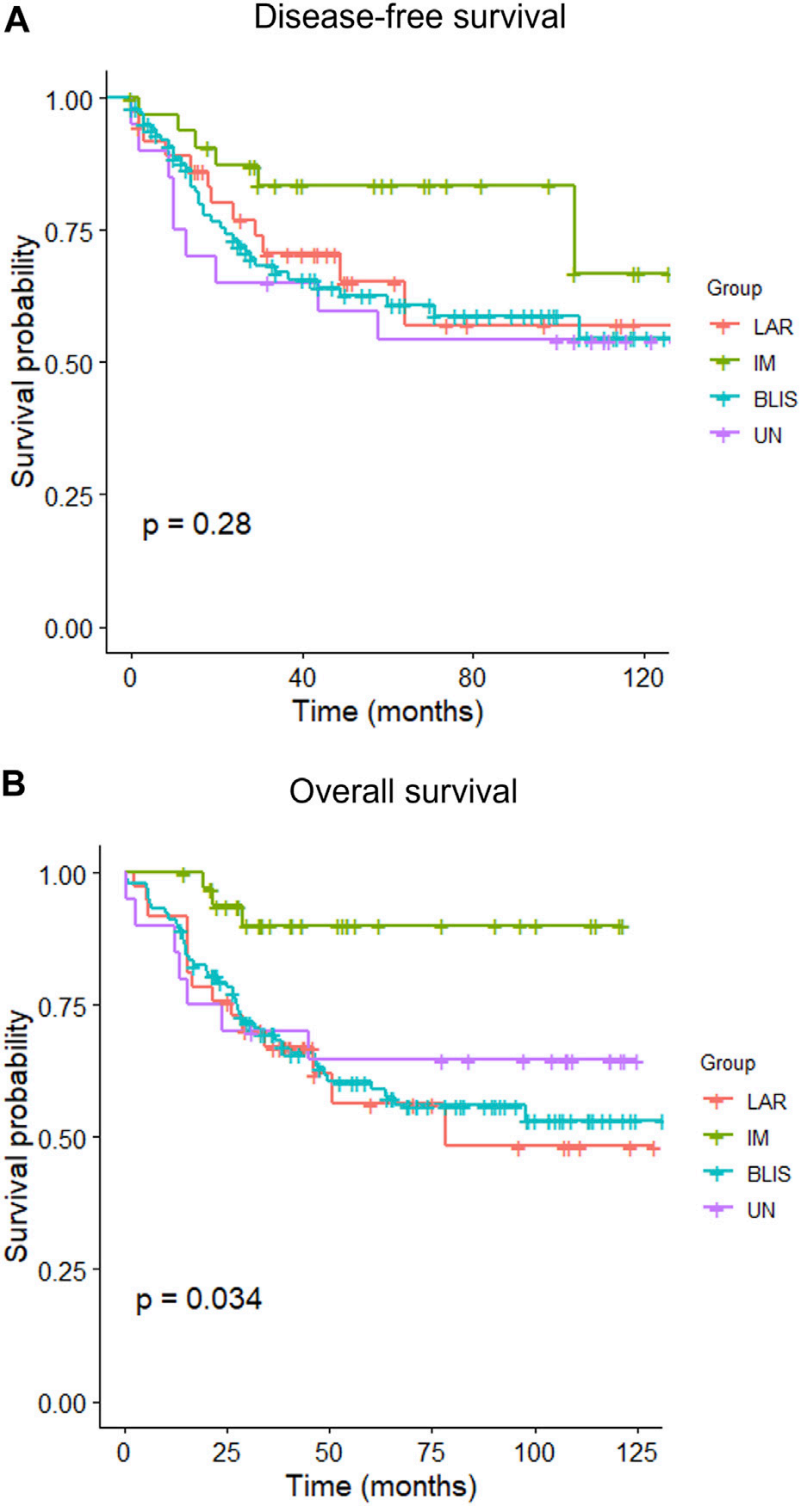
Kaplan–Meier curves of disease-free survival **(A)** and overall survival **(B)** of patients with different IHC-based molecular subtypes of triple-negative breast cancer. BLIS, basal-like immunosuppressed; IM, immunomodulatory; LAR, luminal androgen receptor; UC, unclassifiable.

The median follow-up time was 40.95 months (interquartile range (IQR): 23.48–89.22 months). By the end of the study (December 2021), 63 patients had experienced at least one episode of recurrence, and 66 patients died. The 5-year rate of DFS was 64.7% (95% CI: 0.576–0.726), and OS was 65.0% (95% CI: 0.581–0.728). The Kaplan–Meier estimates of DFS between different IHC-based subtypes did not show any variation (*p* = 0.28), but the OS was significantly different (*p* = 0.034) (Figure 3). MES subtype was not included in the Kaplan-Meier curves because there was only one case. Patients with the IM subtype had a better OS than those with other subtypes, while the survival curves of the patients with other subtypes overlapped. The patient with MES experienced no recurrence and remained alive until the end of the study.

### Prognostic significance of IHC-based TNBC subtypes

Tables 2, 3 present univariate and multivariate Cox regression analyses of DFS and OS, respectively. Age, clinical stage, histologic type, lymphovascular invasion, and radiotherapy were significant factors in the univariate analysis of DFS. However, only age and clinical stage remained significant in the multivariate model.

**TABLE 2:** Univariate and multivariate Cox regression analysis for disease-free survival.

Variables	Univariate analysis		Multivariate analysis	
	HR (95% CI)	*p*-value	HR (95% CI)	*p*-value
Age, years				
<50	1			
≥50	0.55 (0.33–0.9)	0.018	0.45 (0.25–0.81)	0.008
Clinical stage				
I	1			
II	1.26 (0.55–2.86)	0.586	0.98 (0.42–2.27)	0.957
III	5.09 (2.29–11.31)	<0.001	3.83 (1.58–9.3)	0.003
IV	129.75 (40.19–418.89)	<0.001	157.17 (43.63–566.12)	<0.001
Histologic type				
Invasive ductal carcinoma	1		1	
Invasive lobular carcinoma	3.52 (1.27–9.72)	0.015	1.54 (0.51–4.61)	0.441
Metaplastic carcinoma	0 (0– Inf)	0.997	0 (0–Inf)	0.998
Other	0 (0– Inf)	0.996	0 (0–Inf)	0.997
Histologic grade				
1–2	1			
3	0.95 (0.51–1.78)	0.874		
Lymphovascular invasion				
No	1		1	
Yes	1.69 (1.03–2.78)	0.039	1.65 (0.97–2.79)	0.066
Chemotherapy				
No	1			
Yes	0.67 (0.27–1.68)	0.396		
Radiotherapy				
No	1			
Yes	1.88 (1.09–3.22)	0.022	0.97 (0.51–1.83)	0.915
IHC-based molecular subtype				
IM	1		1	
LAR	1.97 (0.74–5.26)	0.174	1.27 (0.46–3.49)	0.642
BLIS	2.21 (0.93–5.25)	0.072	1.28 (0.52–3.13)	0.596
MES	0 (0– Inf)	0.997	0 (0–Inf)	0.996
UC	2.5 (0.89–7.05)	0.083	1.5 (0.51–4.43)	0.467

HR, hazard ratio; IHC, immunohistochemistry; BLIS, basal-like immunosuppressed; IM, immunomodulatory; LAR, luminal androgen receptor; MES, mesenchymal-like; UC, unclassifiable.

**TABLE 3:** Univariate and multivariate Cox regression analysis for overall survival.

Variables	Univariate analysis		Multivariate analysis	
	HR (95% CI)	*p*-value	HR (95% CI)	*p*-value
Age, years				
<50	1			
≥50	0.7 (0.43–1.14)	0.131		
Clinical stage				
I	1		1	
II	6.17 (1.46**–**26.14)	0.013	9.05 (2.08–39.35)	0.003
III	17.62 (4.2**–**73.93)	<0.001	26.33 (6–115.51)	<0.001
IV	81.82 (17.69**–**378.37)	<0.001	105.62 (21.69–514.23)	<0.001
Histologic type				
Invasive ductal carcinoma	1			
Invasive lobular carcinoma	2.6 (0.94**–**7.17)	0.064		
Metaplastic carcinoma	0 (0**–**Inf)	0.996		
Other	0 (0**–** Inf)	0.996		
Histologic grade				
1–2	1			
3	1.09 (0.7–1.97)	0.777		
Lymphovascular invasion				
No	1			
Yes	1.27 (0.77**–**2.09)	0.34		
Chemotherapy				
No	1		1	
Yes	0.27 (0.14**–**0.53)	0.001	0.14 (0.07–0.29)	<0.001
Radiotherapy				
No	1			
Yes	1.3 (0.78**–**2.18)	0.307		
IHC-based molecular subtype				
IM	1		1	
LAR	5.04 (1.46**–**17.4)	0.011	2.86 (0.81–10.04)	0.101
BLIS	4.72 (1.46**–**15.24)	0.01	3.29 (1.01–10.72)	0.048
MES	0 (0**–**Inf)		0 (0–Inf)	0.996
UC	3.83 (0.99**–**14.86)	0.052	3.08 (0.79–12.04)	0.106

HR, hazard ratio; IHC, immunohistochemistry; BLIS, basal-like immunosuppressed; IM, immunomodulatory; LAR, luminal androgen receptor; MES, mesenchymal-like; UC, unclassifiable.

In the Cox regression analysis for OS, clinical stage, chemotherapy, and IHC-based subtype were significant factors. Specifically, compared to IM, LAR (HR: 5.05, 95% CI: 1.46–17.4) and BLIS (HR: 4.72, 95% CI: 1.46–15.24) were significantly associated with poorer OS in univariate analysis. However, only BLIS remained significant in the multivariate model (HR: 3.29, 95% CI: 1.01–10.72).

## Discussion

TNBC subtyping based on relevant biological characteristics may help to guide treatment and predict prognosis. In this study, we evaluated IHC-based TNBC subtypes based on studies conducted in a Chinese cohort [9, 10]. We found that BLIS was the most frequent subtype (52.82%), followed by LAR (18.97%), IM (17.4%), and the very rare MES subtype (0.5%). IHC-based subtype was significantly associated with OS but not DFS. Patients with BLIS had significant unfavorable OS compared to patients with IM subtype.

In 2020, Zhao et al. [10] selected four top proteins from a list of differentially expressed genes that helped classify TNBC into four molecular subtypes from a transcriptomic subtyping study of a Chinese cohort [9]. We assessed the IHC-based TNBC subtypes based on the study by Zhao et al. [10], as the population in this study was also Asian. We used the same IHC markers and immunostaining criteria for comparison. Here, we found a difference in IHC-based subtypes. Our TNBC cohort had a considerably higher percentage of patients with BLIS (52.82%) but a lower percentage of patients with LAR (18.97%) than those reported by Zhao et al. (38.1% for BLIS and 28.6% for LAR) [10]. Different proportions of TNBC molecular subtypes among different populations have also been reported. Jiang et al. [9] applied and compared their mRNA-based TNBC subtypes based on 360 Chinese patients to different populations (Caucasian and African-American) in the Cancer Genome Atlas (TCGA) database. They found BLIS (39%–42%) and IM (24%–32%), and MES (15%–20%) had comparable distribution, while LAR was higher in the Chinese cohort (23%) compared to Caucasian (12%) and African-American (9%). Therefore, a higher proportion of Thai patients with TNBC in the current study had the BLIS subtype and a remarkably lower proportion had the MES subtype than both Western and Chinese populations. A high proportion of Hispanic patients (53.2%) with the BLIS subtype has also been found in a US cohort [16]. This evidence suggests that the differences in the distribution of TNBC subtypes may reflect different genetic backgrounds across races or ethnic groups.

Based on molecular subtyping of the Chinese cohort, CD8 was found to be the top-ranked gene with a high area under the ROC curve (AUC) and a good correlation between mRNA and protein expression [10]. CD8 is a marker for cytotoxic T-cells, an essential component of TILs and tumor immune microenvironment [17]. Our study found that the IM subtype, represented by the presence of stromal CD8^+^ TILs, had a favorable prognosis, and this finding is consistent with other studies [10, 18]. The IM subtype has been shown to have higher expression of genes involved in immune pathways, such as cytokine signaling, immune cell signaling, antigen processing as well as presentation, chemokine signaling pathway, immune signal transduction pathway, and immune response process [6–9]. Immunotherapy with immune checkpoint inhibitors, such as PD-L1 inhibitors, has been used to treat TNBC [19]. Previous studies have shown that PD-L1 expression is associated with high levels of CD8^+^ T cell infiltration in TNBC [20, 21]. Therefore, the evaluation of CD8 expression by IHC in TNBC has an implication in predicting prognosis and guiding treatment strategies.

BLIS was the most frequent subtype in our cohort. It was found in younger women, while the LAR and IM subtypes were found in older women. We did not find significant differences of clinical stage and histologic grade among different subtypes which is consistent to previous studies [10, 16]. However, Kim et al. [22] found that histologic grade 3 was most frequently observed in Basal-like type. This subtype is characterized by the upregulation of cell cycle regulators, activation of DNA repair, and downregulation of immune response genes [8, 9]. These biological mechanisms may explain the proliferated phenotypic features of the BLIS subtype which leads to poor survival outcome.

In this study, patients with the LAR subtype were the oldest compared to those with other subtypes, which is consistent with the previous studies [10, 22, 23]. These studies also found that the patients with the LAR subtype had a lower histological grade, and apocrine morphology; however, we did not find these patterns. In addition, they found that the LAR subtype is associated with a favorable prognosis compared to MES subtype while in our study, the LAR subtype was associated with poorer survival compared to the IM subtype, although it was not statistically significant in multivariate analysis. We did have only one patient with MES subtype, therefore, the results would not be explicitly compared. Consistent with other studies, survival patterns among some different subtypes were overlapped [7, 10]. In our study, the survival pattern of LAR, BLIS, and UC overlapped and in the study by Zhao et al, BLIS, LAR, IM, and UC did not differ [10]. These results indicate that the current surrogate molecular subtyping needs further refinement to improve clinical relevance.

Our study had some limitations. First, we used long-term stored FFPE tissue blocks, which might have affected IHC staining results. Second, we evaluated immunostaining in TMA tissues, which was used as a substitute for the entire tumor. Third, the number of cases, especially for some subtypes, was low and may be insufficient to determine the association of subtypes with clinicopathological characteristics and survival outcomes. Fourth, it is known that gene expressions from transcriptomic data are the gold standard of molecular subtyping, however, we did not have our transcriptomic data to validate the results. Therefore, the findings in this study should be interpreted with caution. Lastly, even though IHC markers have more clinical implications in routine practice than mRNA expression profiles, a unified method of immunostaining evaluation is needed before their usage. In particular, protein markers with cytoplasmic staining, like DCLK1, may pose some challenges than those with nuclear reactivity as staining evaluation is more difficult to quantify.

In conclusion, our study, which is based on the IHC of surrogate markers, revealed certain differences in the distribution of TNBC subtypes in Thai patients compared to that in the Chinese and Western populations. The BLIS subtype accounted for half of the cases, whereas the MES subtype was rare. This evidence suggests that molecular TNBC subtypes may reflect different genetic backgrounds across races or ethnic groups. However, refinement of molecular subtyping of TNBC needs further refinement to improve clinical relevance.

## Data Availability

The raw data supporting the conclusion of this article will be made available by the authors, without undue reservation.
